# Triple Loop Atrial Flutter Occurring After a Lung Transplant

**DOI:** 10.1002/joa3.70297

**Published:** 2026-02-16

**Authors:** Takumi Yamada

**Affiliations:** ^1^ UF Health Cardiovascular Center University of Florida College of Medicine Jacksonville Florida USA

**Keywords:** atrial flutter, catheter ablation, lung transplant, triple loop

## Abstract

High‐density activation mapping with a multipolar catheter revealed a triple loop atrial flutter occurring after a lung transplant in which two electrical connections between the recipient left atrium and the donor left pulmonary vein cuff and a surgical scar at the mitral isthmus acted as a substrate.

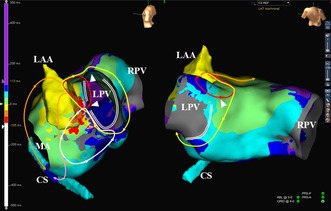

Atrial flutters (AFLs) are common clinical problems that occur after a lung transplant [1, 2]. Some of these AFLs are associated with a surgical scar and an electrical connection (EC) between the donor pulmonary vein (PV) cuff and the recipient left atrium (r‐LA) [1, 2, 3].

A 56‐year‐old woman with atypical AFL occurring 10 years after a bilateral lung transplant due to cystic fibrosis was referred for catheter ablation. At baseline, the patient was in an atypical AFL with a tachycardia cycle length (CL) of 230 ms.

During the AFL, the activation sequence recorded from the coronary sinus (CS) catheter exhibited a “reverse‐C” pattern (Figure 1). Activation mapping using a multipolar mapping catheter (Advisor HD Grid, Abbott Laboratories) revealed a triple loop AFL that shared a common critical isthmus (CI) at the ridge between the recipient left atrail appendage (r‐LAA) and donor left PV (d‐LPV) cuff (Figure 2A and Video S1). The first AFL formed a clockwise reentrant loop using 2 ECs between the d‐LPV cuff and the r‐LA at the upper posterior aspect and the site adjacent to the CI (AFL‐1); the second formed a clockwise reentrant loop around the LPV cuff (AFL‐2); and the third formed a counter‐clockwise reentrant loop around the localized scar at the anterolateral mitral isthmus (AFL‐3). The activations from the AFLs propagated around the mitral annulus in a clockwise as well as counter‐clockwise fashion, reflecting a “reverse‐C” type of atrial activation pattern within the CS (Figure 2). Prolonged fractionated atrial electrograms were recorded around the CI (white circles in Figure 1).

**FIGURE 1:**
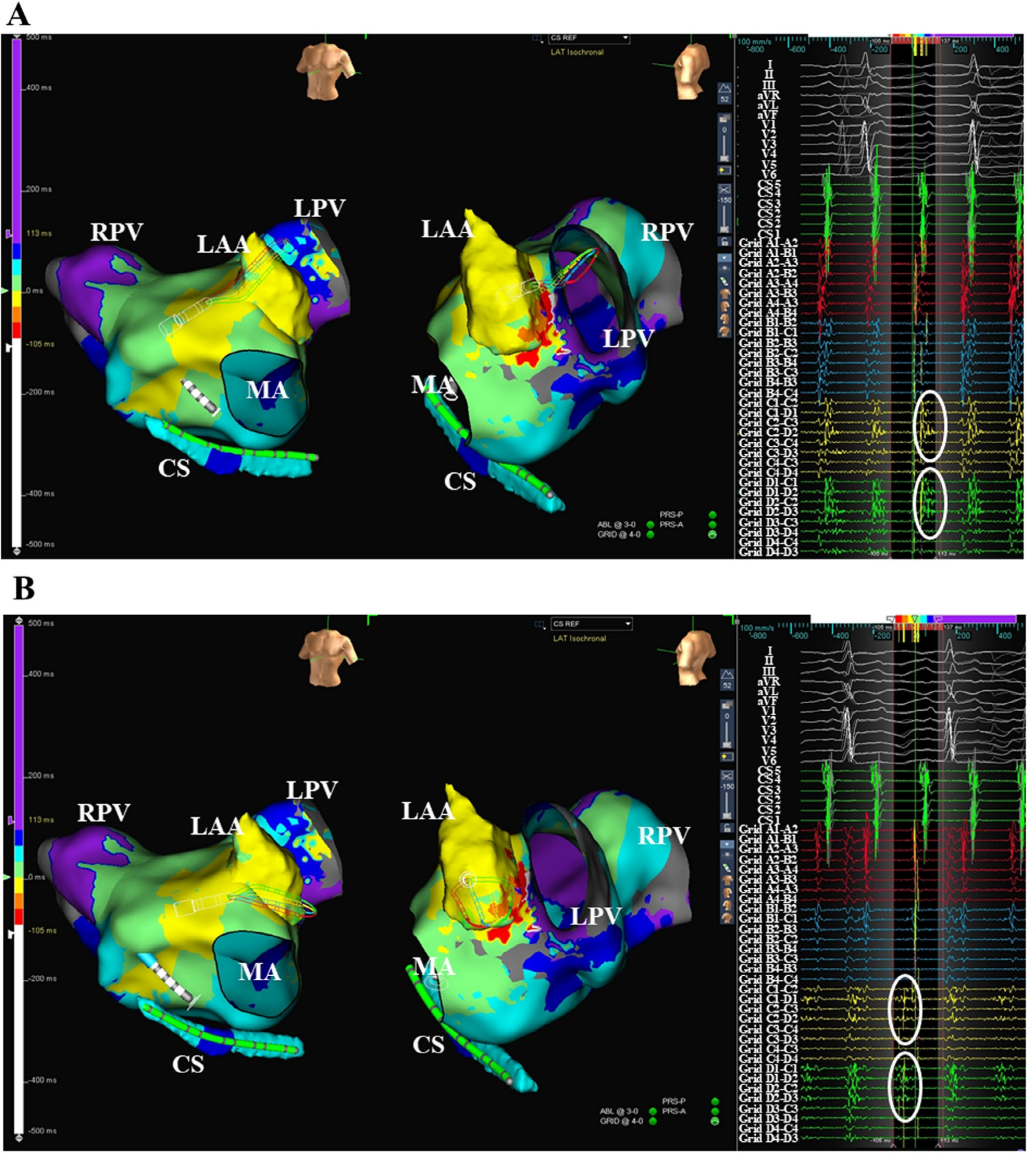
Activation maps of the atrial flutters (AFLs) (left panels) and cardiac tracings recorded from the mapping catheters during the AFLs (right panels). Note that the multipolar mapping catheter was positioned within the left pulmonary vein (LPV) cuff (Panel‐A) and the left atrial appendage (LAA) (Panel B). CS, coronary sinus; CS 1 to 5, the first to fifth electrode pairs of the CS catheter; MA, mitral annulus; RPV, right pulmonary vein.

**FIGURE 2:**
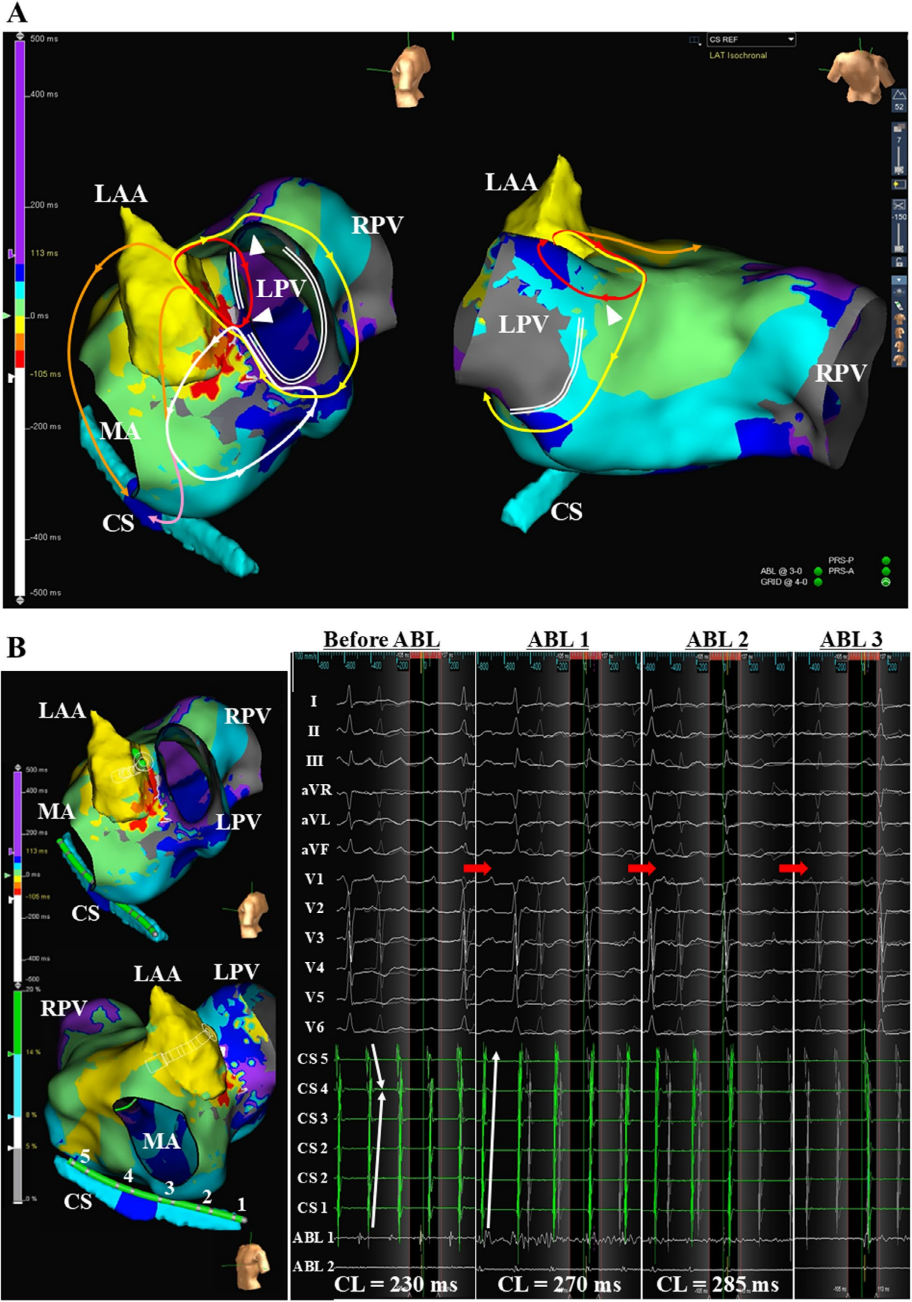
(A) Activation maps were recorded during the AFLs. The double lines indicate a line of conduction block between the LPV cuff and the left atrium. The red, yellow, and white circles indicate the reentrant loops of AFL‐1, AFL‐2, and AFL‐3, respectively. The orange and pink arrows indicate the activations around the mitral annulus, reflecting a “reverse‐C” pattern of the atrial activation sequence within the CS. The radiofrequency ablation was likely to have eliminated the 2 AFLs (red and yellow circles) first, resulting in the disappearance of the activations (orange arrows). Consequently, the remaining activation (pink arrow) was likely to have reflected the “distal to proximal” atrial activation sequence within the CS. The white arrowheads indicate electrical connections between the donor LPV cuff and recipient left atrium. (B) Activation maps exhibiting the catheter positions (left panels) and cardiac tracings exhibiting the changes during the successful ablation (right panels). The abbreviations are as in Figure 1.

Radiofrequency catheter ablation (RFCA) was started at the septal edge of the CI. Soon after that, the AFL CL prolonged and the atrial activation sequence recorded within the CS changed from a “reverse‐C” pattern to a “distal to proximal” pattern (Figure 2B). RFCA was continued across the CI, and the AFL terminated following a further prolongation of the AFL CL, and sinus rhythm was restored (Figure 2B). Following this, multiple radiofrequency applications were delivered across the CI and at the EC between the d‐LPV cuff and upper r‐LA revealed by the voltage map (Figure 3). Remapping performed during sinus rhythm confirmed electrical isolation of the LPV and elimination of the CI (Figure 3).

**FIGURE 3:**
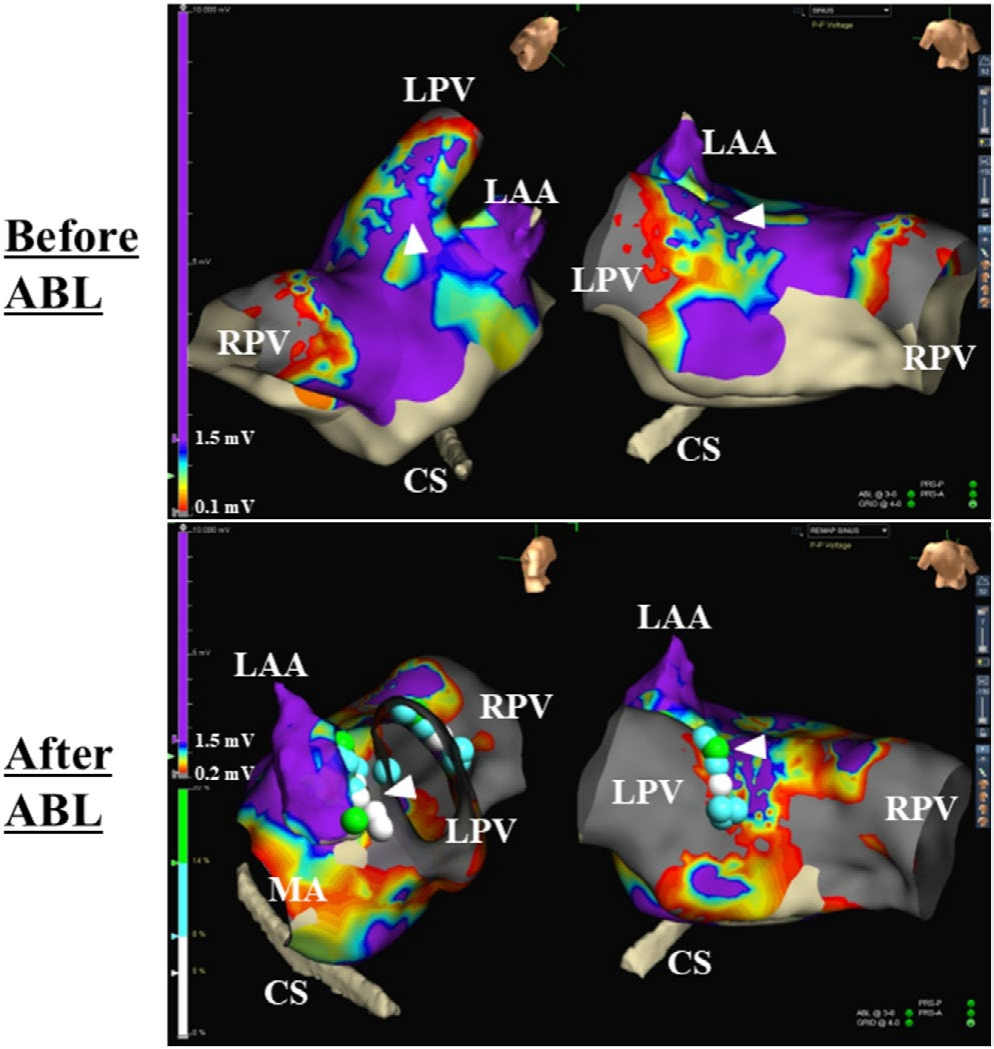
Voltage maps recorded before and after the ablation (ABL). The green, light blue, and white tags indicate the ablation sites. The white arrowheads indicate electrical connections between the donor LPV cuff and the recipient left atrium. The abbreviations are as in Figure 1.

During more than 3 years of follow‐up, the patient has been free of any tachycardias without any antiarrhythmic drugs.

In this case, high‐density activation mapping revealed a triple loop AFL with a critical slow conduction zone at the ridge between the r‐LAA and d‐LPV cuff There were several requirements for this type of AFL to occur. First, two ECs between the d‐LPV cuff and the r‐LA occurred after the lung transplant. Multiple ECs can act as a substrate of multiple loop AFL around the PV cuff [3]. In this case, 2 ECs were involved in the circuit of one of the triple loops of AFL (AFL‐1). Second, there was a surgical scar near the LPV cuff, which served as an anatomical obstacle for one of the triple loops of AFL (AFL‐3). Third, the slow conduction in the smallest reentrant circuit of AFL‐1 provided enough time for the other AFLs (AFL‐2 and AFL‐3) to rotate through their reentrant circuits.

In this case, the initial RFCA targeting the common CI was begun from the side of the AFL‐1 reentrant circuit, and it prolonged the AFL CL (Figure 2B). Simultaneously, the atrial activation sequence within the CS changed from a “reverse‐C” pattern to a distal to proximal pattern. The “reverse‐C” pattern of the atrial activation sequence within the CS reflected that an atrial activation from AFL‐1 had reached the proximal CS earlier than that from AFL‐3 (Figure 2A). Therefore, the change in the atrial activation sequence within the CS was likely to have resulted from the elimination of AFL‐1. These findings suggested that AFL‐1 was likely to have been the dominant reentrant circuit.

This case was unique in that 2 ECs between the r‐LA and d‐LPV cuff and a surgical scar after the lung transplant acted as a substrate of the triple loop AFL, which shared a common CI at the ridge between the r‐LAA and d‐LPV cuff.

## Funding

The author has nothing to report.

## Conflicts of Interest

The author declares no conflicts of interest.

## Supporting information


**Video S1:** A propagation map recorded during the triple loop AFL.

## Data Availability

The data that support the findings of this study are available on request from the corresponding author. The data are not publicly available due to privacy or ethical restrictions.
